# Potentiality of Using Luojia 1-01 Nighttime Light Imagery to Investigate Artificial Light Pollution

**DOI:** 10.3390/s18092900

**Published:** 2018-09-01

**Authors:** Wei Jiang, Guojin He, Tengfei Long, Hongxiang Guo, Ranyu Yin, Wanchun Leng, Huichan Liu, Guizhou Wang

**Affiliations:** 1 Institute of Remote Sensing and Digital Earth, Chinese Academy of Sciences, Beijing 100094, China; jiangwei@radi.ac.cn (W.J.); 17888827308@163.com (H.G.); yinry@radi.ac.cn (R.Y.); lengwch@radi.ac.cn (W.L.); liuhc@radi.ac.cn (H.L.); wanggz01@radi.ac.cn (G.W.); 2 University of Chinese Academy of Sciences, Beijing 100049, China; 3 Key Laboratory of Earth Observation Hainan Province, Sanya 572029, China; 4 Sanya Institute of Remote Sensing, Sanya 572029, China

**Keywords:** Luojia 1-01, nighttime light imagery, NPP/VIIRS, light pollution, human activity

## Abstract

The successful launch of Luojia 1-01 complements the existing nighttime light data with a high spatial resolution of 130 m. This paper is the first study to assess the potential of using Luojia 1-01 nighttime light imagery for investigating artificial light pollution. Eight Luojia 1-01 images were selected to conduct geometric correction. Then, the ability of Luojia 1-01 to detect artificial light pollution was assessed from three aspects, including the comparison between Luojia 1-01 and the Suomi National Polar-Orbiting Partnership Visible Infrared Imaging Radiometer Suite (NPP-VIIRS), the source of artificial light pollution and the patterns of urban light pollution. Moreover, the advantages and limitations of Luojia 1-01 were discussed. The results showed the following: (1) Luojia 1-01 can detect a higher dynamic range and capture the finer spatial details of artificial nighttime light. (2) The averages of the artificial light brightness were different between various land use types. The brightness of the artificial light pollution of airports, streets, and commercial services is high, while dark areas include farmland and rivers. (3) The light pollution patterns of four cities decreased away from the urban core and the total light pollution is highly related to the economic development. Our findings confirm that Luojia 1-01 can be effectively used to investigate artificial light pollution. Some limitations of Luojia 1-01, including its spectral range, radiometric calibration and the effects of clouds and moonlight, should be researched in future studies.

## 1. Introduction

Light pollution refers to the excessive use of artificial light, mainly outdoor lighting [1,2]. Mismanaged artificial light can alter the nighttime sky brightness [1], eclipse natural starlight [2], and disrupt circadian rhythms [3]. Evidence from previous studies have suggested that light pollution can affect animal behaviors [4,5,6], ecological environments [7,8], human health [9,10], and astronomical observations [2,11]. Moreover, light pollution requires excessive power consumption [12], which can waste energy resources and increase greenhouse gas emissions [12,13]. Therefore, the monitoring and assessment of light pollution is essential for maintaining the ecological environment, meeting sustainable development goals and improving the life quality of urban residents.

Compared with ground-based measurements, nighttime light remote sensing is suitable to acquire large-scale and high numbers of artificial lighting data with low costs [14,15,16,17,18,19]. The observations of nighttime light provide an opportunity to understand light pollution [20,21]. Currently, there are two types of commonly used nighttime remote sensing data: the Defense Meteorological Satellite Program Operational Linescan System (DMSP-OLS) [22,23] and the Suomi National Polar-Orbiting Partnership Visible Infrared Imaging Radiometer Suite (NPP-VIIRS) [24,25,26]. These data are widely utilized to analyze urbanization [27,28], estimate socioeconomic parameters [13,29], and assess military conflicts [30,31,32]. The investigations of light pollution at the national [14,33], regional [21], and global [34] scales are documented. However, the DMSP-OLS is not calibrated and it needs systematic correction before applying it to light pollution investigations [14,21]. The advantage of DMSP-OLS is its long-term past observations, but the calibration has to be done with care [34]. Moreover, the NPP-VIIRS imagery with on-board calibration are used to produce the world atlas of artificial sky luminance [1] and to explore the growth rates of lit areas and the total radiance of artificial light at the global scale [34].

**Figure 1:**
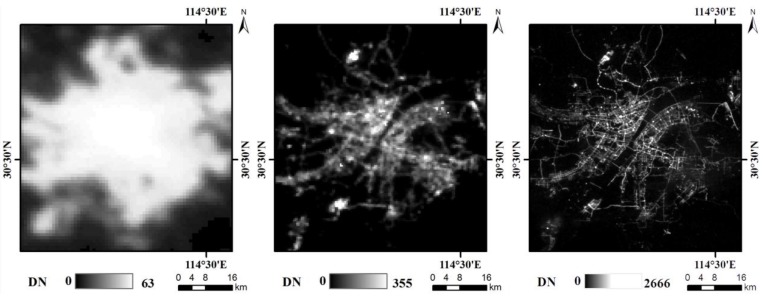
Nighttime imagery from DMSP-OLS, NPP-VIIRS and Luojia 1-01 in Wuhan, China: (**a**) is DMSP-OLS in 2013, (**b**) is NPP-VIIRS in May 2018; (**c**) is Luojia 1-01 on 13 June 2018.

On 2 June 2018, the Luojia 1-01 satellite produced by Wuhan University, which was designed to acquire new high-resolution nighttime light imagery, was launched [35]. The parameter comparisons of DMSP-OLS, NPP-VIIRS and Luojia 1-01 are shown in Table 1. The spatial resolution of the new Luojia 1-01 nighttime light data has greatly improved with on-board calibration. Compared with DMSP-OLS and NPP-VIIRS, the spatial details of artificial light can be clearly observed by Luojia 1-01, which are shown in Figure 1.

The Luojia 1-01 has better spatial resolution than the other two satellite images. However, there is a lack of documentation on the application of Luojia 1-01 nighttime light imagery in assessing light pollution. Therefore, the motivation of this paper is to assess the potential of using Luojia 1-01 nighttime light imagery to assess artificial light pollution. To achieve this goal, we will examine the ability of Luojia 1-01 to detect artificial outdoor lighting, determine the light sources of light pollution, and explore the patterns of urban light pollution. Finally, the limitations of Luojia 1-01 nighttime light imagery are summarized in the discussion section. This study will encourage more researchers to use Luojia 1-01 to investigate light pollution, and the conclusions will help governments make policies to regulate and plan urban light pollution.

## 2. Materials and Methods

### 2.1. Data Source

**Figure 2:**
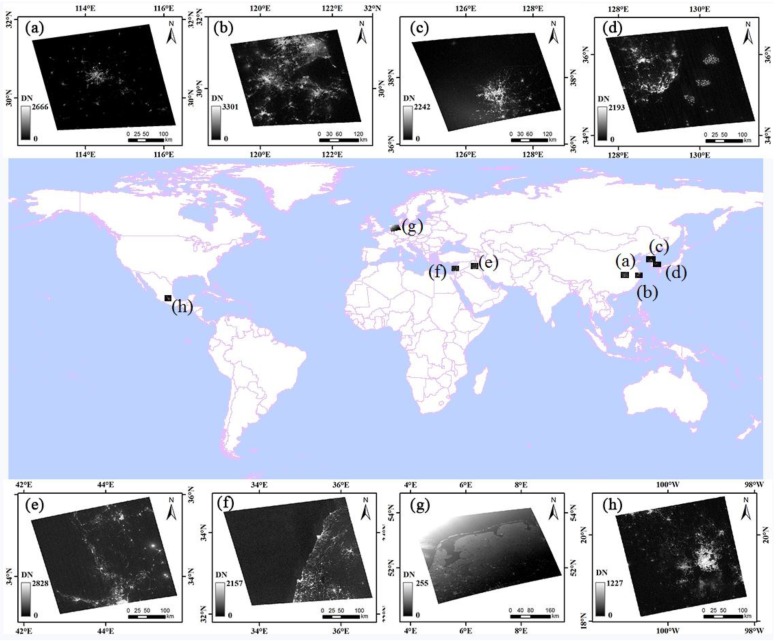
The distribution of the experiment images: (**a**) image of Wuhan in China; (**b**) image of Hangzhou and Shanghai in China; (**c**) image of Seoul in South Korea; (**d**) image of Busan in South Korea; (**e**) image of Baghdad in Iraq; (**f**) image of Haifa in Israel; (**g**) image of Amsterdam in the Netherlands; (**h**) image of Mexico City in Mexico.

High-resolution Luojia1-01 data are freely available for scientists to download from the Hubei data and application center [36]. In this study, several factors, including the cloud cover, lunar cycle, city size, gas flares, and religious buildings, are considered when selecting images for investigation. Figure 2 shows the distribution of the study areas and origin images, and the metadata of the experiment images are summarized in Table 2.

### 2.2. Nighttime Light Imagery Processing

**Figure 3:**
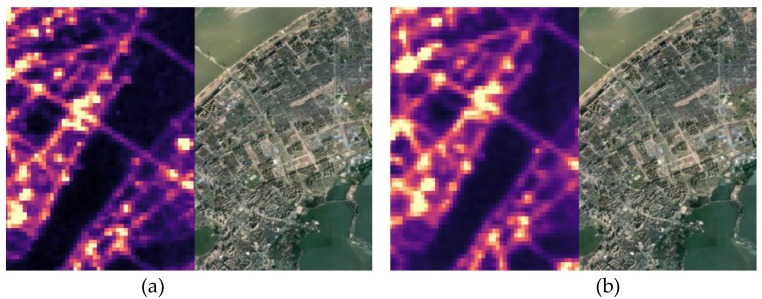
The geolocating performance of LuoJia1-01 nighttime light imagery: (**a**) refers to the performance before ortho-rectification; (**b**) refers to the performance after geometric correction.

High-precision geometric positioning is the basis of remote sensing applications [37]. According to a released report of LuoJia1-01 [35], the released images are systematically geometric corrected, and the positioning accuracy ranges from 0.49 km to 0.93 km. Consequently, before new products with improved geolocation accuracy are released, precise geometric correction is necessary for LuoJia1-01 to accurately investigate artificial light pollution. To accurately investigate artificial light pollution, these experimental images need to be geometrically corrected. Because the spatial resolution of LuoJia1-01 nighttime light imagery is high, the road network can be clearly observed. Thus, accurate ground control points (GCPs) can be collected using road intersections. For each image scene, approximately 50 GCPs were manually collected to estimate the rational function coefficients (RPCs) using the L1-norm-regularized least squares method [38], which are used to conduct ortho-rectification with the help of the global Shuttle Radar Topography Mission(SRTM) Digital Elevation Model (DEM) data. The geolocating performance of LuoJia1-01 nighttime light imagery in Wuhan is shown in Figure 3. In Figure 3a, the geometric error in the bridge can be clearly found. Figure 3b shows that the geometric position accuracy can be significantly improved after ortho-rectification. Through nighttime light imagery processing, the geometric positioning error of the experimental images can be reduced.

## 3. Results

### 3.1. The Ability of Luojia 1-01 to Detect Artificial Outdoor Lighting

**Figure 4:**
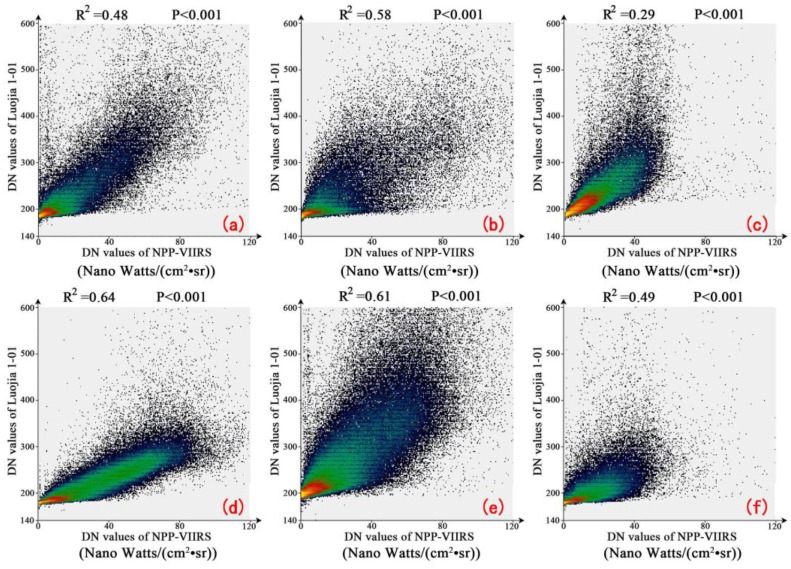
The scatter plot between Luojia 1-01 and Suomi National Polar-Orbiting Partnership Visible Infrared Imaging Radiometer Suite (NPP-VIIRS) for six cities: (**a**) refers to Busan; (**b**) refers to Haifa; (**c**) refers to Hangzhou; (**d**) refers to Mexico City; (**e**) refers to Seoul; (**f**) refers to Wuhan.

**Figure 5:**
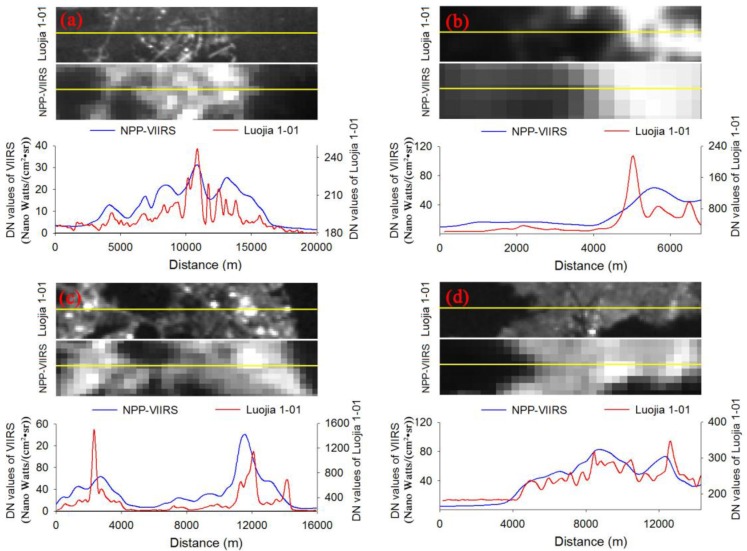
Latitudinal transects of the nighttime light DN between LuoJia1-01 and Suomi National Polar-Orbiting Partnership Visible Infrared Imaging Radiometer Suite (NPP-VIIRS): (**a**) refers to Wuhan; (**b**) refers to Hangzhou; (**c**) refers to Seoul; (**d**) refers to Mexico City.

To investigate the artificial light pollution, the ability of Luojia 1-01 to detect nighttime outdoor lighting should be first assessed. Six cities, including Wuhan, Hangzhou, Seoul, Busan, Haifa and Mexico City, are selected as the study areas. First, the digital number (DN) range of Luojia 1-01 and NPP-VIIRS across these cities are calculated, as shown in Table 3. The result shows that Luojia 1-01 can detect a higher dynamic range than NPP-VIIRS. Second, to compare the consistency of luminary artificial light observed by Luojia 1-01 and NPP-VIIRS, the Luojia 1-01 images of six cities are resampled with the spatial resolution of NPP-VIIRS at nighttime. The scatter plots regarding to two types of nighttime light data are shown in Figure 4. From the correlation coefficient, the spatial consistency between Luojia 1-01 and NPP-VIIRS is high in Seoul and Mexico. However, the different image acquisition dates and spectral sensitivity can reduce the consistency. The ability of Luojia 1-01 nighttime light imagery to acquire detailed information on urban luminaires is evaluated by comparing the latitudinal transects of the DN between Luojia 1-01 and NPP-VIIRS in four cities. The results are shown in Figure 5. The DN values of two types of data increase towards the urban core and decrease away from the urban core. Moreover, the variability of Luojia 1-01 in the core urban areas is higher than that of NPP-VIIRS. This result suggests that Luojia 1-01 can capture finer spatial details of artificial nighttime light compared with NPP-VIIRS nighttime light images.

### 3.2. Determination the Source of Artificial Light Pollution

**Figure 6:**
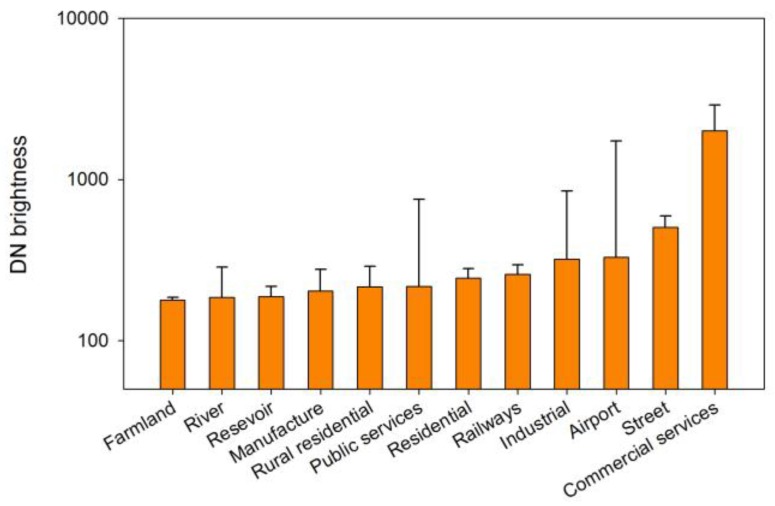
Digital number (DN) brightness for each land use type.

The LuoJia1-01 nighttime light satellite can capture a higher dynamic range and finer spatial details of luminaires. Thus, it can be used to determine the source of artificial light pollution. First, we investigate the artificial light pollution sources within different land use types. The land use type classification is referenced in Levin’s work [39]. The typical land use boundaries were manually drawn by using high-resolution Google Earth satellite imagery, based on which the averages of the nighttime brightness for each type were calculated, and the results are shown in Figure 6. A clear difference in the average brightness can be found between various land use types. The high-brightness artificial light pollution areas were located in the commercial services, streets, airports, industrial areas and public services, while dark areas were associated with farmland, rivers, reservoirs and rural residential areas.

**Figure 7:**
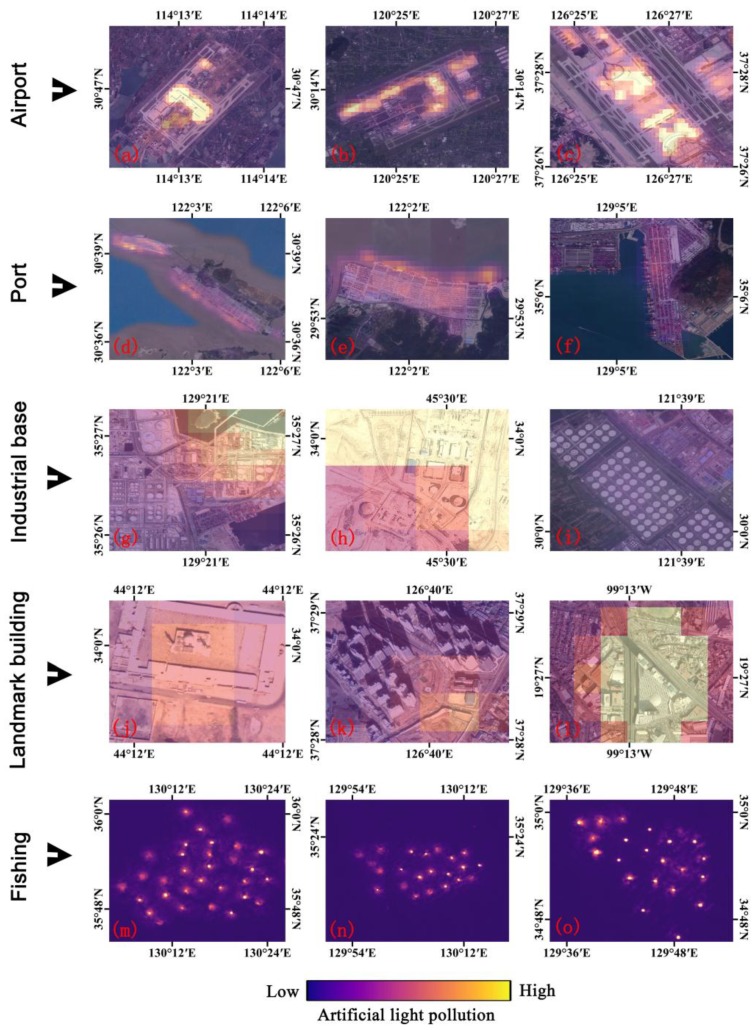
Validation of the high artificial light pollution with high-resolution images: (**a**–**c**) represent high artificial light pollution in airports; (**d**–**f**) represent high artificial light pollution in ports; (**g**–**i**) represent high artificial light pollution in industrial bases; (**j**–**l**) represent high artificial light pollution in landmark buildings; (**m**–**o**) represent high artificial light pollution regarding fishing. (**a**–**l**) are the LuoJia1-01 nighttime light images overlaid on Google Earth high-resolution images with 50% transparency. (**m**–**o**) are LuoJia1-01 nighttime light images.

Furthermore, the high-resolution images were employed to validate high artificial light pollution. Figure 7a–c show that the luminary brightness in airports and terminals are brighter than those on airport runways. As to the ports, the artificial light in lifting areas is brighter than that in container areas (Figure 7d–f). The artificial light pollution in industrial bases (Figure 7g–i) have a high level. Some landmark buildings, including mosques (Figure 7j), high-density residential areas (Figure 7k), and commercial buildings (Figure 7l) are sources of high artificial light pollution in urban areas. These sources of light pollution tend to be near the urban core areas, which may be detrimental to human health [10,40]. Moreover, the luminaires from marine fishing can also be captured by LuoJia1-01(Figure 7m–o), which can be used to monitor the spatial distribution of fishing activity [41,42] and assess the disturbance of artificial light pollution on the marine ecosystem [43].

**Figure 8:**
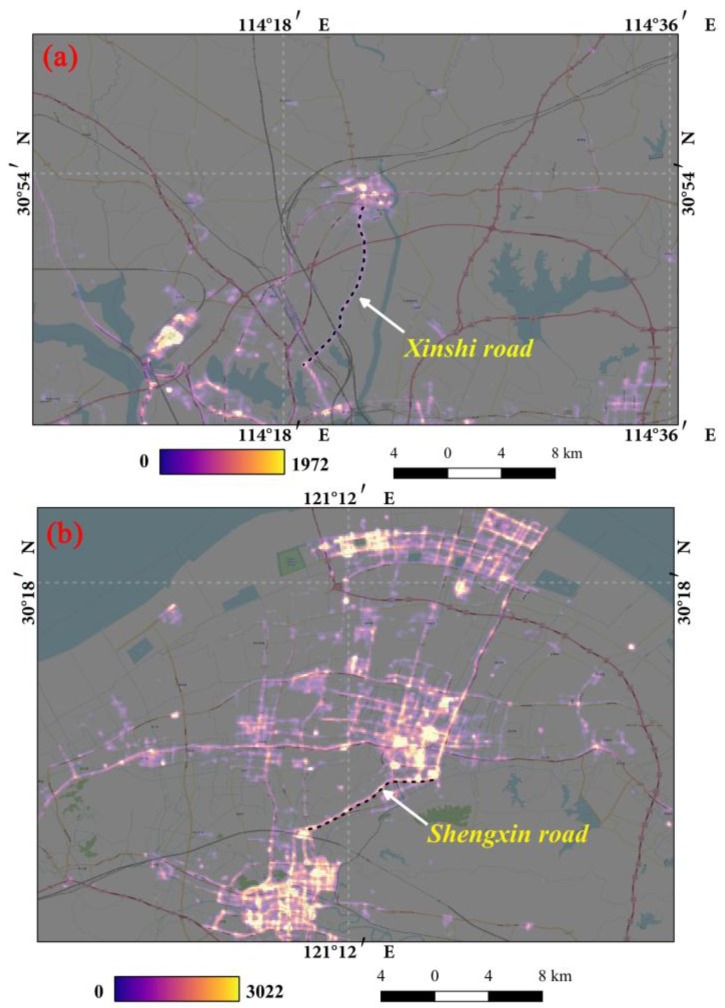
The artificial nighttime light along roads: (**a**) refers to the Xinshi road in Wuhan; (**b**) refers to the Shengxin road in Ningbo. (**a**) and (**b**) are the LuoJia1-01 nighttime light images overlaid on Google Earth high-resolution images with 50% transparency.

**Figure 9:**
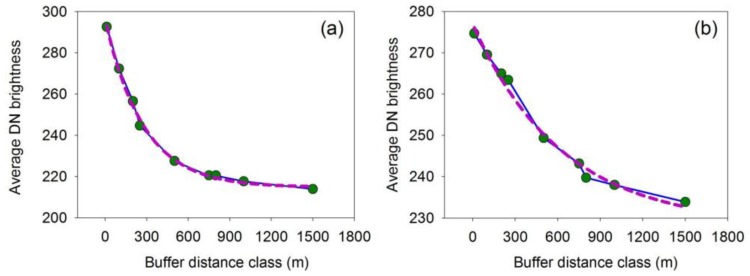
Average luminary brightness within multiple buffers: (**a**) refers to Xinshi road in Wuhan; (**b**) refers to Shengxin road in Ningbo.

Among the various land use types, artificial light along roads is the major source of light pollution [44]. The high-resolution LuoJia1-01 nighttime light imagery provides the potential to detect the artificial light along roads. Figure 8 shows the road networks with nighttime light imagery in Wuhan and Ningbo. To investigate the light pollution regarding the road light, multiple buffer analysis is employed on the main roads (Xinshi road and Shengxin road). Figure 9 shows that the average DN brightness decreases away from the road. This result is because high-brightness lights are widely used for traffic, while the light is of low brightness around residential areas and open spaces.

### 3.3. Exploration the Patterns of Urban Light Pollution

**Figure 10:**
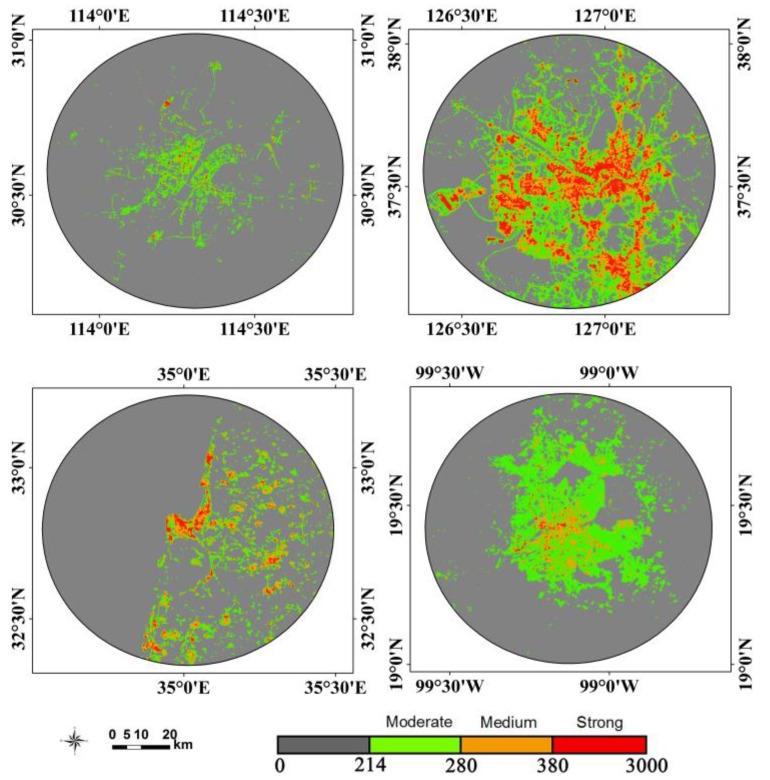
Urban light pollution patterns: (**a**) refers to Wuhan; (**b**) refers to Seoul; (**c**) refers to Haifa; (**d**) refers to Mexico City.

**Figure 11:**
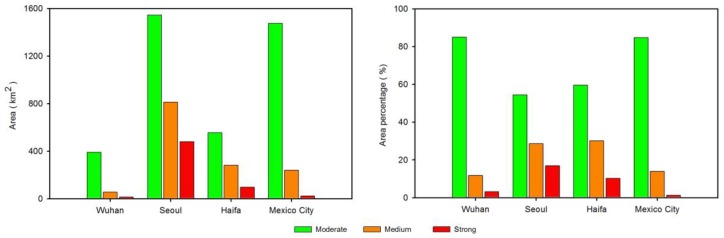
The areas and area percentages of different light pollution levels.

**Figure 12:**
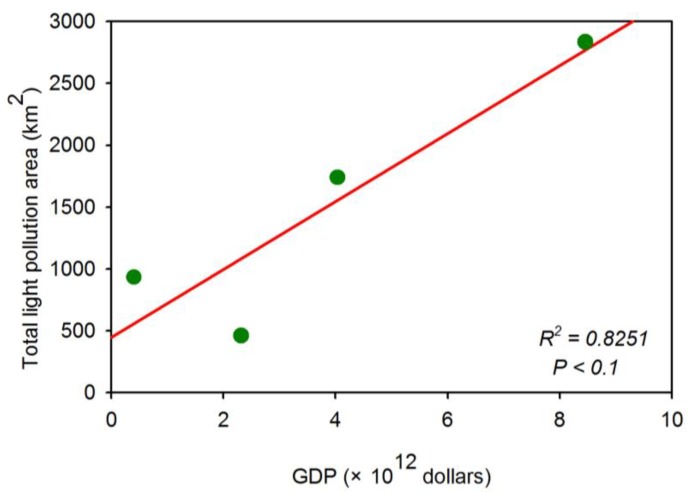
The correlation between the total light pollution area (refers to three light pollution level areas) and gross domestic product (GDP).

Urban light pollution patterns relate to the economic development levels, populations, and energy consumption [45]. Four cities, including Wuhan, Seoul, Haifa and Mexico City, were selected to explore the patterns of urban light pollution. The nighttime light imagery within a 45 km buffer is classified with different levels using the natural breaks (Jenks) method [46]. Figure 10 suggests that the light pollution patterns of four cities were significantly different and the light pollution decreased away from the urban cores. Moreover, the areas and area percentages regarding the different light pollution levels are illustrated in Figure 11. For the area corresponding to moderate light pollution, the top one is Seoul, which is followed by Mexico City. Compared with strong light pollution, the area of Seoul was larger than that of Mexico. As to the area percentages, the patterns of Haifa and Seoul are similar. Compared with Haifa and Seoul, the area percentages of Wuhan and Mexico City regarding the moderate light pollution are high. Moreover, the correlation between the total light pollution area (refering to three light pollution level areas) and gross domestic product (GDP) is shown in Figure 12. Although the urban sample is only four cities, this result suggests that the light pollution is highly related to economic development.

## 4. Discussion

Over the past 20 years, DMSP-OLS and NPP-VIIRS were the main data sources in the field of nighttime light remote sensing [26,47]. Some studies documented the new high-resolution nighttime light imagery, such as JL1-3B [48] and EROS-B [39], but these data are not freely available for acquisition [39]. Nighttime light imagery from International Space Station (ISS) is open access data with high spatial resolution, however, these images lack automated georeferencing. The successful launch of Luojia 1-01 complements the existing nighttime light data with a high spatial resolution of 130 m. This paper is the first study to investigate the potential of using Luojia 1-01 nighttime light imagery to evaluate light pollution. Compared with NPP-VIIRS, the Luojia 1-01 can capture a higher dynamic range and finer spatial details of luminaires. Moreover, we investigated the light pollution within different land cover types and found that the light pollution is significantly different between various land use types. The source of high-brightness light pollution was determined with high-resolution images, and the average DN brightness decreased away from the road. Finally, the urban light pollution pattern in four cities was explored and the result suggested that the light pollution was highly related to the level of economic development. 

**Figure 13:**
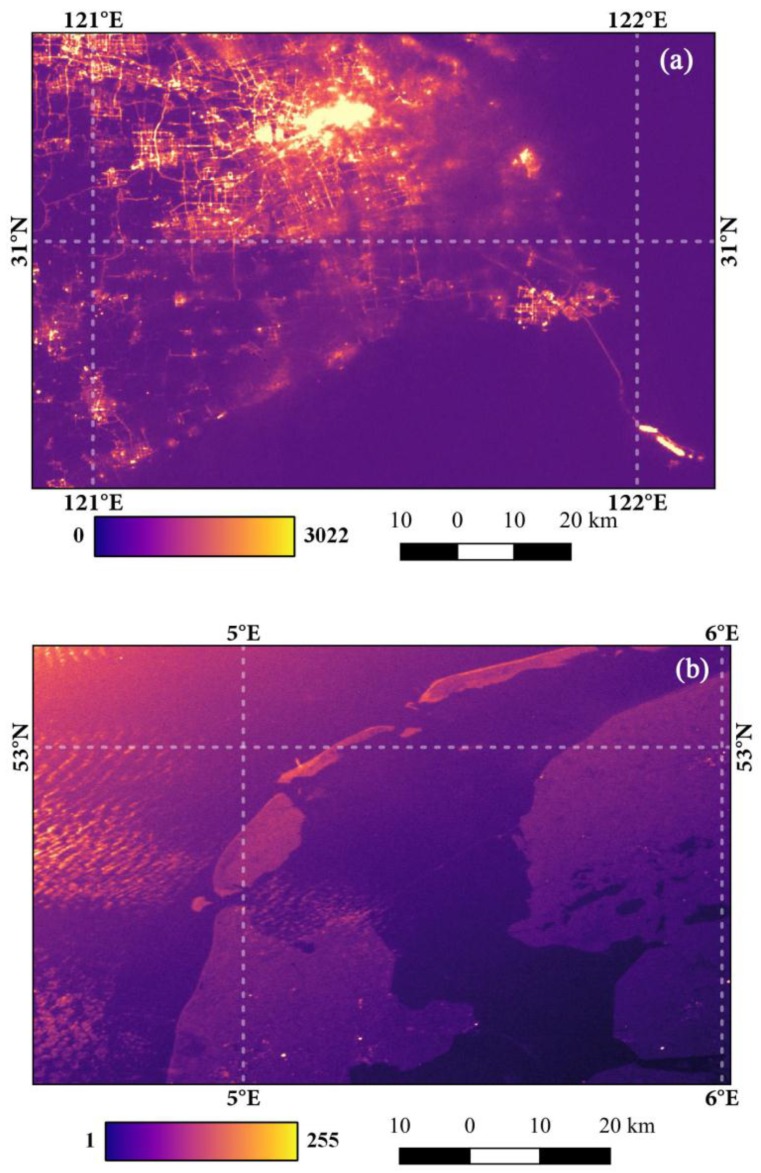
The quality of Luojia 1-01 nighttime light imagery with clouds and a full moon: (**a**) refers to the nighttime light imagery with clouds in Shanghai, China; (**b**) refers to the nighttime light imagery with a full moon near Amsterdam, the Netherlands.

Although this study confirmed that Luojia 1-01 can be applied to investigate artificial light pollution, there are still some limitations for Luojia 1-01. First, “white” LEDs are widely used for street lighting and the commonly used high correlated color temperature (CCT) white light-emitting diodes (LEDs) emit a large fraction of radiation in the range of 450 nm to 480 nm [34,39,49,50]. However, the spectral range of Luojia 1-01 can detect from only 480 μm to 800 μm [35]. Thus, some radiance information of LED luminaires will not be captured by the Luojia 1-01 nighttime light satellite. This limitation is a challenge when the Luojia 1-01 is used in estimating electricity, energy and carbon emissions. Second, Luojia 1-01 lacks accurate ground-based measurements to conduct the radiometric calibration [19,51]. Thus, Luojia 1-01 cannot be directly compared with NPP-VIIRS nighttime light imagery. This limitation will make it difficult to quantify urban night light pollution [49]. Third, clouds and moonlight can affect the quality of Luojia 1-01 nighttime light imagery. Figure 13a shows the imagery with clouds in Shanghai, China. In the thin cloud area, artificial light is attenuated by clouds. The lunar calendar of Figure 13b is 16 May with a full moon and the sea and land near Amsterdam is lighted. The radiance in the urban area contains nighttime light and moonlight. Future studies can be carried out on the preprocessing of Luojia 1-01 nighttime light imagery to eliminate the effects of clouds and moonlight. 

**Figure 14:**
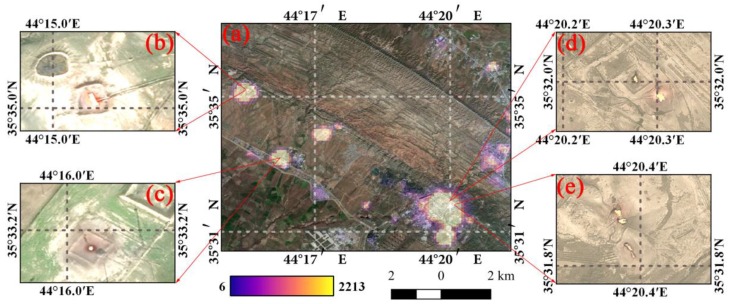
The gas fire captured by Luojia 1-01 nighttime light imagery in Iraq: (**a**) is the nighttime light image overlaid on a Google Earth high-resolution image with 50% transparency; (**b**–**e**) are the gas fires shown in Google Earth high-resolution images.

Due to the high sensitivity of Luojia 1-01, the LuoJia1-01 nighttime light satellite can capture a higher dynamic range. In addition to nighttime lighting, LuoJia1-01 can also clearly detect gas fires in oilfields. Figure 14 shows the high-brightness DN caused by gas fires in Iraq. Therefore, the LuoJia1-01 nighttime light imaging can be used to detect gas fires and estimate oil and gas production [52] in future studies.

## 5. Conclusions

Luojia 1-01 is a new satellite that can be used to acquire high-resolution nighttime light imagery at a global scale. Compared with NPP-VIIRS nighttime light imagery, the Luojia 1-01 nighttime light imagery shows great potential as a future light pollution data source with an improved spatial resolution. This paper is the first study to investigate the potential of Luojia 1-01 nighttime light imagery in artificial light pollution. The conclusions are summarized as follows. 

(1) Compared with NPP-VIIRS, the spatial consistency between Luojia 1-01 and NPP-VIIRS is high, and Luojia 1-01 can detect a higher dynamic range and capture the finer spatial details of artificial nighttime light.

(2) The averages of the artificial light brightness were different between various land use types. The high-brightness artificial light pollution areas were located in commercial services, streets, airports, industrial areas, public services and marine fishing, while the darkest areas were associated with farmland, rivers, reservoirs and rural residential areas. Moreover, the average luminary brightness decreased away from roads.

(3) The light pollution patterns of four cities were significantly different and the light pollution decreased away from the urban core. The areas with moderate light pollution are Seoul and Mexico City, and the strong light pollution area in Seoul was larger than that in Mexico City. Compared with Seoul and Mexico City, the areas in Wuhan and Haifa corresponding to the three light pollution levels were smaller. This result suggested that the light pollution was related to economic development.

This study provides evidence for the advantages of using Luojia 1-01 nighttime light imagery and confirms that it can be effectively used to investigate artificial light pollution. These conclusions can help researchers understand the artificial light pollution using Luojia 1-01 high nighttime light imagery. However, the spectral range, radiometric calibration and the effects of clouds and moonlight are still limitations for the widespread use of Luojia 1-01 nighttime light imagery. These shortcomings will be the future research directions.

## Figures and Tables

**Table 1:** The parameter comparisons of Defense Meteorological Satellite Program Operational Linescan System (DMSP-OLS), the Suomi National Polar-Orbiting Partnership Visible Infrared Imaging Radiometer Suite (NPP-VIIRS), and Luojia 1-01.

Satellite	DMSP-OLS	NPP-VIIRS	Luojia 1-01
Operator	U.S. Department of Defense	NASA/NOAA	Wuhan University
Available years	1992–2013	December 2011–present	June 2017–present
Wavelength range	400–1100 μm	505–890 μm	480–800 μm
Orbital altitude	830 km	830 km	645 km
Orbit	Polar orbit satellite	Polar orbit satellite	Polar orbit satellite
Spatial resolution	2.7 km	742 m	130 m
Width	3000 km	3000 km	260 km
Revisit time	12 h	12 h	15 d
Pixel saturated	Saturated	No saturated	No saturated
On-board calibration	No	Yes	Yes

**Table 2:** The description of experiment data used in this study.

Study Area	File Name	Acquisition Date	Lunar Calendar	Cloud Cover	Covered City
a	LuoJia1-01_LR201806145301_20180613144138_HDR_0024_gec	13 June 2018	30 April	Cloud free	Wuhan
b	LuoJia1-01_LR201806175049_20180616141538_HDR_0016_gec	16 June 2018	3 May	Some clouds	Hangzhou and Shanghai
c	LuoJia1-01_LR201806193121_20180618132805_HDR_0011_gec	18 June 2018	5 May	Cloud free	Seoul
d	LuoJia1-01_LR201806158490_20180614132921_HDR_0002_gec	14 June 2018	1 May	Some clouds	Busan
e	LuoJia1-01_LR201806057936_20180604191551_HDR_0019_gec	4 June 2018	21 April	Cloud free	Baghdad
f	LuoJia1-01_LR201806273072_20180622195500_0013_gec	22 June 2018	9 May	Cloud free	Haifa
g	LuoJia1-01_LR201806304569_20180629211025_HDR_0058_8bit	29 June 2018	16 May	Some clouds	Amsterdam
h	LuoJia1-01_LR201806057936_20180605045718_HDR_0000_gec	5 June 2018	22 April	Some clouds	Mexico City

**Table 3:** The digital number (DN) range of Luojia 1-01 and Suomi National Polar-Orbiting Partnership Visible Infrared Imaging Radiometer Suite (NPP-VIIRS) in six cities.

Study Area	DN Range of Luojia 1-01	DN Range of NPP-VIIRS (Nano Watts/(cm^2^·sr))
**Busan**	162–3952	0.39–243.66
**Haifa**	172–2745	0.23–266.52
**Hangzhou**	156–3887	0.71–207.11
**Mexico City**	160–2580	0.54–150.64
**Seoul**	141–2894	0–528.57
**Wuhan**	163–1972	0.16–355
